# Syrian refugees in Lebanon: the search for universal health coverage

**DOI:** 10.1186/s13031-016-0079-4

**Published:** 2016-06-01

**Authors:** Karl Blanchet, Fouad M. Fouad, Tejendra Pherali

**Affiliations:** Public Health in Humanitarian Crises Group, London School of Hygiene & Tropical Medicine, London, UK; American University of Beirut, Beirut, Lebanon; University College London Insitute of Education, London, UK

## Abstract

The crisis in Syria has forced more than 4 million people to find refuge outside Syria. In Lebanon, in 2015, the refugee population represented 30 % of the total population. International health assistance has been provided to refugee populations in Lebanon. However, the current humanitarian system has also contributed to increase fragmentation of the Lebanese health system. Ensuring universal health coverage to vulnerable Lebanese, Syrian and Palestinian refugees will require in Lebanon to redistribute the key functions and responsibilities of the Ministry of Health and its partners to generate more coherence and efficiency.

## Background

The crisis in Syria, which has finished its fifth year in March 2016, continues to take a devastating toll on the country’s civilian population. An estimated 7.5 million people are displaced within Syria, and more than 4 million refugees have fled to neighbouring countries including, 1.2 million Syrians refugees in Lebanon registered with the United Nations High Commission for Refugees [28]. As a result, the number of people residing in Lebanon has increased sharply by at least 30 % since March 2011 in a country of just 4 million Lebanese [28]. Health care, water and sanitation facilities, shelter, and other resources that were already strained in Lebanon have been put under further pressure due to the sudden influx Syrian refugees.

## Objectives

This paper seeks to analyse the shortcomings of the Lebanese health system and propose structural alternatives to cater for the needs of diverse groups of population currently living in Lebanon.

## Conclusion

This paper highlight some of the existing challenges to ensure universal health coverage of Syrian refugees and the disadvantaged groups of the Lebanese population and explore the disjuncture between humanitarian response and crisis in the health system in Lebanon.

### A dispersed population

Based on the policy of the Lebanese Government to ban the construction of refugee camps [8, 23], the majority of Syrian refugees live in host communities across 1,600 localities in Lebanon, mainly concentrated in the poorest regions of Lebanon, Bekaa and north Lebanon [28]. These refugees live in various types of shelters, including rented rooms, apartments, garages, and in some areas, in dilapidated tents on land rented from private landowners. Living conditions of these families are alarming, lacking in access to potable water or sanitation facilities (for around 30 % of refugees) [13]. Beyond access to basic services including, primary health care and education for school-aged children, many refugees fleeing Syria have serious health care needs due to, amongst other things, pre-existing chronic conditions and injuries suffered during the conflict [6, 20, 26]. However, on arriving in Lebanon they are met with an overstretched system in which the services available to refugees are limited and difficult to access. The poorest communities in northern Lebanon, which were already struggling to access basic services, have now received the most vulnerable Syrian refugees [12]. The scattered and spontaneous settlements of refugee population across the country have stern effects on existing health services.

### A fragmented health system

Lebanon, like many countries in the Middle East such as Syria is experiencing an epidemiological transition with an increasingly ageing population suffering from chronic and non-communicable diseases [22]. Lebanon has a fragmented and uncoordinated health care system, which is highly privatized and based on user fees [17]. This expenditure is concentrated in high cost curative technological interventions, and the number of Lebanese individuals making use of primary health care centers remains limited and the quality of services varies by region and provider [17]. One of the structural weaknesses in the Lebanese health care system is related to the fact that the role of the Ministry of Health has focused almost exclusively on the provision of services, while its role in prevention, planning and regulation remains limited. This is particularly true in light of the expanding role of the private sector [24]. This has required the health system in Lebanon to adapt to the changing health needs of the population [7, 24, 34].

The main challenges in the current health system are summarized by the unclear policy and strategy for health care on the part of the government and the overwhelming preponderance of an unregulated private sector in financing and provision of health care [2]. The high out-of-pocket expenditures leading to exposure of households to financial risks from ill health and the minimal public expenditure on primary health care compared to secondary and tertiary care bring more burdens on the Lebanese and the vulnerable population in particular [24].

After the civil war, Lebanon’s healthcare system was built on sectarian standards. Currently, religious charities and sectarian parties play a critical role in providing primary health care. About 28 % of medical centers and dispensaries are run by Christian and Muslin charities. Another 15 % of basic health care come from political parties [5].

Multiple actors deliver the main source of funding for the health care system, mainly through social and private insurance schemes [25]. For uninsured Lebanese citizens accounting for 50 % of the Lebanese population [21], the Ministry of Public Health provides services as the last resort, either through public hospitals or contracted private hospitals, and covers 95 and 85 % of hospital care costs consecutively and 100 % of medication costs for chronic and high-risk diseases [1, 10]. Primary health care is provided through a network of centres, supported by the Ministry of Public Health and the Ministry of Social Affairs [16, 17]. The centres are predominately run by NGOs through contractual agreements between the Ministry of Public Health and the NGO. The Ministry of Public Health procures essential drugs and vaccines for the centres, which then charge a fee of LP 15,000 (approximately US$10) per visit. Hospital and more specialized care are mostly provided by the private sector, with 86 % of hospital beds privately owned and the remaining 14 % in the public sector [10]. Prior to the refugee crisis, Lebanon’s Ministry of Public Health had a sizeable budget deficit, with delayed payments to contracted private hospitals as a result. According to the World Bank, the outstanding payments to private hospitals from public purchases is estimated at US$800 million, with significant burden on the financial system of many hospitals as a result [32]. This situation has further exacerbated due to the influx of Syrian refugees, who constitute almost one-third of the people living in Lebanon [28].

### A fragmented humanitarian system

The arrival of 1.2 million refugees in Lebanon has constituted a new challenge for the Lebanese health system. At first, the humanitarian system led by UNHCR was in charge of the health and protection of Syrian refugees. In May 2013, due to the high costs of healthcare in Lebanon and reduction of international funding, UNHCR had to increase out of pocket payment for refugees from 15 to 25 %.UNHCR has adopted a public health approach which prioritizes affordable and accessible basic primary health and emergency care, over costlier and complex treatments and hospital care, with the aim of ensuring coverage for the greatest number of refugees in Lebanon. Syrian refugees in Lebanon have access to primary health care across Lebanon, which is largely provided by UNHCR’s non-governmental organisations (NGO) partners, in addition to the state provision maintained by the Ministry of Social Affairs. Those refugees registered with UNHCR and between the ages of five and 60 can get access to health care in the centres managed by NGOs for a fee of LP 3,000 to LP 5,000 per consultation (approximately US$2 to US$3). Individuals also have to pay for x-rays and other diagnostic tests, which are required for referral to hospital for further treatment or medicines, where these are needed. For those deemed vulnerable, 85 % of diagnostic costs are covered by UNHCR and 100 % of the cost of medicines under a programme provided for by a Lebanese charitable organization. This includes those under five years and over 60 years of age, disabled people, pregnant women and nursing mothers. Refugees who require other kinds of care, such as hospital care (secondary and tertiary care), must be referred by a primary health care centre that works with the UNHCR system, with the exception of a life-threatening emergency where this is not possible. For those that meet UNHCR’s criteria for hospital care, 75 % of the treatment costs are covered, with the remaining 25 % - as well as the cost of medicines - to be covered by the individual, unless they meet UNHCR’s vulnerability criteria or are victims of torture or sexual or gender-based violence, in which case 100 % of costs are covered [10].

Notably, the Lebanese health system also needs to cover the vulnerable Lebanese groups (336,000 living with less than $2.4 per day), Lebanese returnees from Syria (50,000), Palestinian refugees from Syria (70,000) and Palestinian refugees in Lebanon (335,000) [12]. As a result, the Lebanese health system is fragmented and structured by target group (See Table 1). Each social group of the population has access to different services with a different level of social protection.

**Table 1:** The fragmented structure of the Lebanese health system by population group in 2016 (framework adapted from Londoño and Frenk [19])

*Functions of health system*	Population groups					
	*Non-poor*		*Vulnerable*			
	Socially insured	Privately insured	Vulnerable Lebanese	Palestinian in Lebanon	Palestinian refugees from Syria	Syrian refugees
*Stewardship*	MoH	Private sector	MoH	UNRWA	UNRWA	UNHCR
*Financing*	National Social Security Fund (NSSF)	Insurance premiums	Taxes	International	International	International
*Delivery*	Public sector	Private sector	Public services	Humanitarian sector	Public services, NGO and private sector	Public services NGO and private sector

### An inequitable health system

In this segmented model of health system, the choice of provider and patient pathway is actually not determined by patients’ choice but by the social group classification. Each social group has different rights and level of access to healthcare services. The level of financial protection for refugees from Syria is precarious and the provision of healthcare obliges the already indigent refugees to privately bear the costs [21].

The humanitarian sector has largely failed to address the basic health needs of the populations and the social protection system is not adequate to reduce poverty amongst refugees [21, 29]. While such a failure could partially be explained by the lack of international funding, the inequitable structure of the health system can explain the discrepancies between population needs and service coverage. It is estimated that 82 % of Syrians registered with UNHCR as refugees pay rent and 55 % of Syrians registered as refugees have debt larger than $400 [4]. The 25 % contribution to healthcare fees requested for each patient irrespective of their financial situation jeopardises health care needs of the poor refugees [15]. A recent survey report by UNHCR [27] reveals that 24 % of Syrian refugees had returned to Syria since registering with UNHCR and 11 % had returned to see medical care despite the ongoing risks of violent conflict.

Nevertheless, responding to basic health needs to Syrian refugee population is not necessarily a matter of choice for the Lebanese authorities as the failure to provide assistance could lead to social tensions between the two communities. There are also risks of epidemics (i.e. measles, polio and cholera) in the areas of congested settlements and the authorities need to reduce pressure on local systems (e.g. health facilities, schools, municipalities) [33].

On the other hand, Lebanese returnees, who most of them had been living in Syria for decades, face conditions very similar to Syrian refugees. More than 70 % of them had not benefited from any health assistance and paid for all health related services [14].

As Lebanese citizens, they are qualified to the health services provided by the Lebanese Ministry of Public Health (MOPH) facilities. However, this is not always the case, because they are often unaware of, or unfamiliar with, the system [14].

### Towards universal health coverage

Similar to what Londoño and Frenk [19]) described for Latin America, Lebanon is currently facing challenges both on the demand and supply of health services. On the demand side, refugee populations have been experiencing challenges caused by epidemiologic and demographic dynamics: a high burden of non-communicable diseases due to ageing populations and lifestyles combined with a burden of common diseases related to poor hygiene and lack of access to basic health services [31, 34]. On the supply side, the health facilities have experienced cost escalation of service provision and with the financial incapacity to cover all the needs of the refugee populations, who themselves do not have access to social protection schemes.

As highlighted in the 2016–2020 strategy on non-communicable diseases, universal health coverage is at the heart of the vision of the actors on the health system in Lebanon [30]. Lebanon has to find its own path to be able to achieve universal health coverage in a very pluralistic health system. A fragmented health system structure, as it currently is in Lebanon, has proven in the past to be inequitable and inefficient [9]. For each social group, the health system is composed of various sub-health systems with their specific stewardship and financing systems. Each steward is in a situation of monopoly where the choice and expectations of patients are not considered. The only group of population who has the full power and rights to choose their health care providers are the Lebanese populations who are privately insured.

While as challenging politically it might be, it is now important for the survival of the health system in Lebanon to integrate the refugees (Palestinian and Syrian) and the vulnerable Lebanese population into a single health system [3]. This would help reduce inequities and inefficiencies and enhance coherence between the public, private as well as the humanitarian sectors. The new health strategy of Lebanon integrates universal health coverage as a key principle. It requires for the health system to address both related to the redefinition of the benefit package, which currently does not include primary health care, and define the maximum of financial burden that can be supported by each category of the Lebanese society [3]. It is estimated that reinforcing primary health care would cost the Government an additional 6 % of the current national health budget [3]. As seen in neighbouring countries, the humanitarian sector can contribute to the roll out of the Lebanese universal health coverage plan and can contribute both to vulnerable Lebanese and Syrian refugees.

The example from UNHCR that convinced Iran to undertake a health insurance scheme that would provide over one million Afghan refugees with a level of access to secondary and tertiary care that is similar to that of an “average” Iranian, is an important one that can bring the idea of universal health coverage to be applied to displaced populations [11].

Another example comes from the Iraqi refugees’ crisis after 2003 when more than 2 million Iraqi had to flee to the neighbouring countries escaping from war, UNHCR developed an Exceptional Care Committee that assesses individual cases and makes objective decisions about the referral based primarily on prognosis and cost [18].

One solution for integrating the refugees in the health system in Lebanon, is to build a new model that Frenk entitled “structured pluralism” where the State would have a lead role in terms of stewardship to define quality standards and policies, regulate the competition framework to ensure fairness and transparency and allocate resources according to population’s needs [9] (See Table 2). The health system would not be defined by population groups but by health system functions. This would help integrate the large humanitarian system into the Lebanese health system while guaranteeing efficiencies as well as enhancing capacities and transparency in the governance of the health system [25] (Table 2).

**Table 2:**
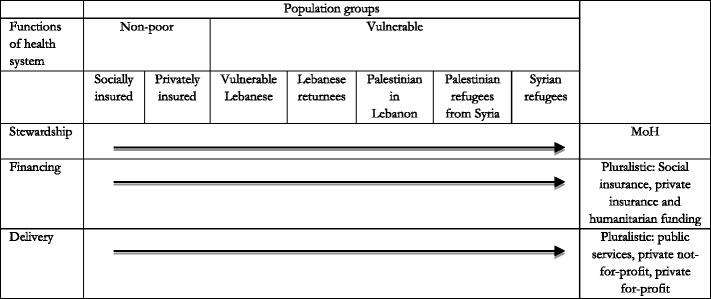
A structured pluralistic health system (Adapted from Londoño and Frenk [19]))

The idea of this new structure is to put back the patient at the centre of the health system and make the state authorities more responsible for health services while increasing their stewardship in the delivery of public services. Health needs are universal and fundamental human rights, which should not depend on socioeconomic capabilities of the people. In order to achieve egalitarian health care, state regulated health system is necessary where health care is regarded as the basic tenet of human society in which geopolitical boundaries are irrelevant in defining access and quality of care. The patient is not anymore a receiver of services but has the right to receive high quality care from the best providers. The universal health coverage is the matter of global concern and therefore, a strong consolidated health system is needed to provide the best health care that people of Lebanon including, Syrian refugees deserve.

## References

[1] Ammar W."Health Beyond Politics". WHO, MPH, ISBN 978-9953-515-489, Beirut. January 2009.

[2] Ammar W (2009). Health Beyond Politics.

[3] Ammar W (2010). Towards Universal Health Coverage: Universal Community Health Coverage for Preventive and Essential Outpatient Care.

[4] Amnesty International (2014). Agonizing choices: Syrian refugees in need of health care in Lebanon.

[5] Cammett M (2011). Partisan activism and access to welfare in Lebanon. Stud Comp Int Dev.

[6] Coutts A, Fouad FM, Abbara A, Sibai AM, Sahloul Z, Blanchet K (2015). Responding to the Syrian health crisis: the need for data and research. Lancet Respir Med.

[7] El-Jardali F, Merhi M, Jamal D, Dumit MG (2009). Assessment of nurse retention challenges and strategies in Lebanese hospitals: the perspective of nursing directors. J Nurs Manag.

[8] European Commission (2016). Lebanon: Syria crisis.

[9] Frenk J (2015). Leading the way towards universal health coverage: a call to action. Lancet.

[10] Government of Lebanon and the United Nations 2015. Lebanon Crisis Response Plan 2015-16. Beirut, Lebanon

[11] Guterres A, Spiegel P (2012). The state of the world’s refugees: adapting health responses to urban environments. JAMA.

[12] Inter-agency coordination lebanon (2015). Basic Assistance Sector - Mid Year Dashboard June 2015.

[13] Inter-agency coordination lebanon (2015). WASH Sector - Mid-Year Dashboard June 2015.

[14] IOM (2014). Refugees at home - A livelihoods assessment of Lebanese returnees from Syria.

[15] Johns hopkins University Bloomberg School of Public health & Médecins Du Monde 2015. Syrian refugee and Affected Host Population - Health Access Survey in Lebanon. Beirut, Lebanon

[16] Kassak KM, Ghomrawi HM, Osseiran AMA, Kobeissi H (2006). The providers of health services in Lebanon: a survey of physicians. Human Resources for Health..

[17] Kronfol NM (2006). Rebuilding of the Lebanese health care system: health sector reforms. East Mediterr Health J.

[18] Leaning J, Spiegel P, Crisp J (2011). Public health equity in refugee situations. Confl Health.

[19] Londoño JL, Frenk J (1997). Structured pluralism: Towards an innovative model for health system reform in Latin America. Health Policy.

[20] Maziak W, Rastam S, Mzayek F, Ward K, Eissenberg T, Keil U (2007). Cardiovascular health among adults in Syria: a model from developing countries. Ann Epidemiol.

[21] MSNA TEAM (2014). Inter-agency multi-sector needs assessment.

[22] Pierre louis AM, Ayodeji Akala F, Karam H (2014). Public Health in the Middle East and North Africa: Meeting the Challenges of the 21st Century.

[23] Rainey V (2015). No formal refugee camps for Syrians.

[24] Salti N, Chaaban J, Raad F (2010). Health equity in Lebanon: a microeconomic analysis. Int J Equity Health.

[25] Sfier R (2002). Strategy for National Health Care Reform in Lebanon.

[26] Taleb Z, Bahelah R, Fouad F, Coutts A, Wilcox M, Maziak W (2014). Syria: health in a country undergoing tragic transition. Int J Public Health.

[27] UNHCR (2014). Health access and utlisation survey among non-camp Syrian refugees.

[28] UNHCR. 2015. Syria Regional Refugee Response - Inter-agency Information Sharing Portal [Online]. Geneva: UNHCR. Available: http://data.unhcr.org/syrianrefugees/regional.php [Accessed August 20th 2015 2015].

[29] Verme P, Gigliarano C, Wieser C, Hedlund K, Petzoldt M, Santacroce M (2016). The Welfare of Syrian Refugees : Evidence from Jordan and Lebanon.

[30] WHO (2016). Non communicable diseases prevention and control plan (NCD-PCP) Lebanon 2016-2020.

[31] WHO STEPS (2010). Chronic Disease Risk Factor Surveillance. Data Book for Lebanon.

[32] World BANK (2015). Lebanon economic monitor, April 2015: the economy of new drivers and old drags. Lebanon Economic Monitor.

[33] World Health Organisation (2014). WHO warns that progress towards eliminating measles has stalled.

[34] Yamout R, Adib SM, Hamadeh R, Freidi A, Ammar W (2014). Screening for cardiovascular risk in asymptomatic users of the primary health care network in Lebanon 2012-2013. Prevent Chronic Dis.

